# Optimizing Radiation Dose and Image Quality in Stroke CT Protocols: Proposed Diagnostic Reference Levels for Multiphase CT Angiography and Perfusion Imaging

**DOI:** 10.3390/diagnostics14242866

**Published:** 2024-12-20

**Authors:** Robert Forbrig, Christoph G. Trumm, Paul Reidler, Wolfgang G. Kunz, Konstantinos Dimitriadis, Lars Kellert, Johannes Rückel, Thomas Liebig, Robert Stahl

**Affiliations:** 1 Institute for Diagnostic and Interventional Neuroradiology, LMU University Hospital, LMU Munich, Marchioninistr. 15, 81377 Munich, Germany; robert.forbrig@med.uni-muenchen.de (R.F.); christoph.trumm@med.uni-muenchen.de (C.G.T.); johannes.rueckel@med.uni-muenchen.de (J.R.); thomas.liebig@med.uni-muenchen.de (T.L.); 2 Radiologie Augsburg Friedberg ÜBAG, Hermanstraße 15, 86150 Augsburg, Germany; 3 Department of Radiology, LMU University Hospital, LMU Munich, Marchioninistr. 15, 81377 Munich, Germany; paul.reidler@med.uni-muenchen.de (P.R.); wolfgang.kunz@med.uni-muenchen.de (W.G.K.); 4 Department of Neurology, LMU University Hospital, LMU Munich, Marchioninistr. 15, 81377 Munich, Germany; konstantin.dimitriadis@med.uni-muenchen.de (K.D.); lars.kellert@med.uni-muenchen.de (L.K.)

**Keywords:** brain, computed tomography angiography, ischemic stroke, perfusion imaging, radiation exposure

## Abstract

Objective: In suspected acute ischemic stroke, it is now reasonable to expand the conventional “stroke protocol” (non-contrast computed tomography (NCCT), arterial CT angiography (CTA), and optionally CT perfusion (CTP)) to early and late venous head scans yielding a multiphase CTA (MP-CTA) to increase diagnostic confidence. Diagnostic reference levels (DRLs) have been defined for neither MP-CTA nor CTP. We therefore present dosimetry data, while also considering image quality, for a large, unselected patient cohort. Methods: A retrospective single-center study of 1790 patients undergoing the extended stroke protocol with three scanners (2× dual-source, DSCT; 1× single-source, SSCT) between 07/21 and 12/23 was conducted. For each sequence, we analyzed the radiation dose (volumetric CT dose index (CTDIvol); dose length product; effective dose); objective image quality using manually placed regions of interest (contrast-to-noise ratio (CNR)); and subjective image quality (4-point scale: 1 = non-diagnostic, 4 = excellent). The DRL was defined as the 75% percentile of the CTDIvol distribution. The Kruskal-Wallis test was used initially to test for overall equality of median values in each data group. Single post-test comparisons were performed with Dunn’s test, with an overall statistical significance level of 0.05. Results: Dosimetry values were significantly higher for SSCT (*p* < 0.001, each). Local DRLs ranged between 37.3 and 49.1 mGy for NCCT, 3.6–5.5 mGy for arterial CTA, 1.2–2.5 mGy each for early/late venous CTA, and 141.1–220.5 mGy for CTP. Protocol adjustment (DSCT-1: CTP) yielded a 28.2% dose reduction. The highest/lowest CNRs (arterial/early venous CTA, respectively) were recorded for SSCT/DSCT-2 (*p* < 0.001). Subjective image quality was rated excellent except for slightly increased MP-CTA noise at DSCT-2 (median = 3). Conclusions: Our data imply that additive MP-CTA scans only yield a minor increase in radiation exposure, particularly when using DSCT. CTP should be limited to selected patients.

## 1. Introduction

In patients with suspected acute ischemic stroke (AIS), computed tomography (CT) is frequently performed as a primary imaging measure because it represents a broadly available, quick, and cost-effective diagnostic tool when compared to magnetic resonance imaging which, however, has a higher sensitivity for small infarcts [1]. The conventional stroke protocol includes non-contrast CT (NCCT) and single-phase CT angiography (SP-CTA) of the supra-aortal arteries [2,3]. In AIS patients with large vessel occlusion (LVO) of the anterior circulation (e.g., M1 segment of the middle cerebral artery (MCA)) and 6–24 h of presenting with symptoms, additional CT perfusion (CTP) is recommended before opting for mechanical thrombectomy according to the 2019 American Heart Association guidelines [3]. CTP is helpful to identify both the infarct core and penumbra (i.e., the tissue at risk) [4,5,6].

Apart from the abovementioned, it may be reasonable to expand the stroke protocol via dedicated, multiphase CTA (MP-CTA) scans [3,7,8,9,10,11,12]. Methodically, after acquiring NCCT and (arterial) SP-CTA, two identical head CTs are added, yielding the early and late venous phase [10]. By means of this MP-CTA, a respective collateral score allows us to quantify the grade of retrograde pial filling in AIS-LVO. This score correlates well with the final infarct and thus serves as a valuable decision-making tool for the appropriate acute therapy [3,10]. Furthermore, MP-CTA is superior to conventional SP-CTA in both estimating the thrombus length [11] and diagnosing medium vessel occlusion (MVO; e.g., M2/M3-MCA segments) [13].

Regarding the radiation dose of NCCT and SP-CTA, national diagnostic reference levels (DRLs) are continuously updated by the respective federal authorities [14,15]. However, to date, corresponding DRLs have neither been proposed for MP-CTA nor for CTP.

In recent years, several authors have reported dosimetrics either focusing on individual scans (e.g., NCCT alone, SP-CTA alone, or CTP alone) or the conventional stroke protocol [16,17,18,19], while elaborated dosimetry data on the extended stroke protocol including additive MP-CTA scans are scarce [10]. Furthermore, it remains unclear what impact different CT scanner models might have both on radiation exposure and on image quality in this specific CT protocol.

This monocentric CT study therefore aimed to investigate a large, unselected patient cohort in whom the extended stroke protocol was performed in order to introduce novel DRLs, particularly considering MP-CTA and CTP. These DRLs may serve as a dosimetry landmark, allowing for the comparison and optimization of respective protocol settings with respect to the ALARA (as low as reasonably achievable) principle. Furthermore, we compared in-depth dosimetrics and image quality for three contemporary CT scanners (2× dual-source, DSCT; 1× single-source, SSCT) before/after CTP protocol adjustment to enhance generalizability and also implemented a dose-reduced CTP protocol.

## 2. Materials and Methods

### 2.1. Patients

**Figure 1:**
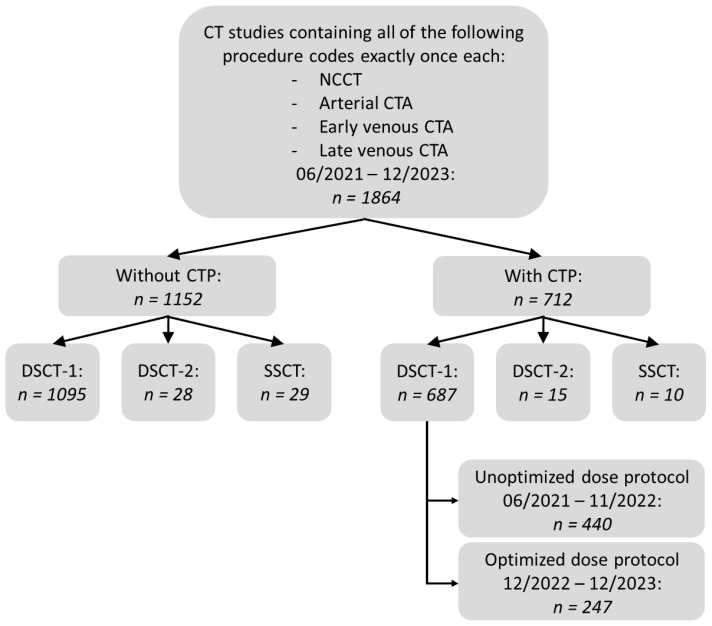
Patient selection. CT, computed tomography; CTA, CT angiography; CTP, CT perfusion; DSCT, dual-source CT; n, number of examinations; NCCT, non-contrast CT; SSCT, single-source CT.

The local ethics committee approved this retrospective study (number 23-0631). The Health Insurance Portability and Accountability Act standards for the privacy of personal health information, as well as the ethical principles of the Declaration of Helsinki, were followed. To define the study population, a radiology information system (RIS) database analysis was carried out, yielding a total of 1864 CT examinations, each of which included an NCCT and MP-CTA between June 2021 and December 2023. In 712 of these cases (38.2%), additional CTP was conducted. The distribution of examinations among the three CT scanners is shown in Figure 1.

### 2.2. Extended Stroke CT Protocol Settings

Details of the individual scanner settings are illustrated in Supplementary Table S1. At our institution, the extended stroke protocol is immediately performed after neurological emergency assessment and primarily carried out using a third-generation DSCT scanner (DSCT-1: 2 × 192-slice SOMATOM Force, Siemens Healthineers, Erlangen, Germany) [20,21] located at the emergency department, which allows for whole-brain CTP. If this CT scanner is not available, the abovementioned protocol is also applied with two other CT scanners (DSCT-2: 2 × 128-slice SOMATOM Drive; SSCT: 128-slice SOMATOM AS+; both Siemens Healthineers).

Regarding the workflow, NCCT is followed by arterial CTA in the caudocranial direction using the bolus tracking technique (CARE Bolus, Siemens Healthineers). In detail, a region of interest (ROI) is manually placed within the aortic arch, and the CT scan starts to automatically move towards the vertex after the intra-arterial density reaches the trigger threshold of 100 Hounsfield units (HUs). Contrast attenuation is achieved by the intravenous administration of 50 mL of non-ionic iodinated contrast material (iomeprol; Imeron^®^ 400 mg I/mL, Bracco Imaging, Konstanz, Germany) at a flow rate of 5 mL/s followed by a 40 mL saline flush. For the completion of MP-CTA, two further identical head CT scans in the caudocranial direction are added with a delay of 8 s each, yielding the early and late venous CTA phase. Then, after the interdisciplinary discussion of the imaging findings between the treating neurologist and neuroradiologist, CTP is only added if clinically indicated. CTP is conducted using 40 mL of the same contrast agent (flow rate 6 mL/s, 100 mL saline flush) with a scan length of 201 mm (DSCT-1), 114 mm (DSCT-2), and 118 mm (SSCT), respectively.

Both automated exposure control (AEC) [22,23] and iterative reconstruction (IR) [24] were enabled for NCCT and MP-CTA. The tube voltage ranged between 100 and 120 kV for NCCT and between 70 and 80 kV for MP-CTA and CTP, respectively.

Regarding DSCT-1, CTP was performed until November 2022 with a dose protocol set by the manufacturer (“unoptimized”), and after December 2022, it was performed with a new protocol (“optimized”). The fixed tube current time product for CTP was set to 250 mAs/180 mAs (DSCT-1, before and after dose optimization) and 200 mAs (DSCT-2 and SSCT), respectively.

### 2.3. Radiation Dose Metrics

Radiation dose structured reports of the examinations were transferred from the picture archiving and communication system to a local open-source dose management system (OpenRem, Version 0.10.0, URL: https://openrem.org/, accessed on 15 February 2024). For radiation dose analysis, we documented the commonly applied dose descriptors of the volumetric CT dose index (CTDIvol) and dose length product (DLP). The effective dose (ED) was calculated by multiplying the DLP with the corresponding conversion factor for the head (0.0016 mSv/mGy∙cm) and/or neck (0.0058 mSv/mGy∙cm) CT according to Deak and colleagues [25]. The local DRL (CTDIvol) was defined as the 75% percentile of the dose distribution [26].

### 2.4. Objective Image Quality Analysis

Out of the whole retrospective dataset (1864 examinations), 20 NCCT and 20 MP-CTA examinations, respectively, were randomly picked for each CT scanner. For CTP analysis, we selected 40 (DSCT-1; unoptimized/optimized period: *n* = 20, each), 15 (DSCT-2), and 10 (SSCT) examinations, respectively. Then, ROIs with a range of 1–3 mm^2^ (vessels) and 15–30 mm^2^ (brain and muscles), respectively [19], were manually placed, while carefully avoiding focal signal inhomogeneities such as calcifications or bone artifacts, within the following regions:

- NCCT: gray matter (GM), white matter (WM) [27];
- MP-CTA: internal carotid artery (ICA), deep WM, superior sagittal sinus (SSS) [16];
- CTP, signal peak phase (core data): ICA, temporal muscles [16,19].

Measurements were repeated thrice for each scan and anatomical structure, and respective mean HU values (i.e., signal) and standard deviations (i.e., noise) were documented. Signal-to-noise (SNR) and contrast-to-noise (CNR) values were then calculated using the following formulas:

- SNR = signal/noise;
- CNR = (mean vascular signal − background signal deep WM or temporal muscles)/image noise.

### 2.5. Subjective Image Quality Analysis

Two experienced neuroradiologists with 12 (R.F.) and 8 years (R.S.) of experience in diagnostic neuroradiology assessed, in consensus, 10 randomly picked examinations for each CT scanner and documented the image quality by applying a 1–4 scale (1, severe image noise, non-diagnostic; 2, moderate image noise, sufficient visualization of GM/WM, vessels and cerebral perfusion; 3, minor image noise, good visualization of GM/WM, vessels and cerebral perfusion; and 4, low image noise, excellent visualization of GM/WM, vessels and cerebral perfusion). Collaterals were rated according to Menon et al. (good, intermediate, and poor) [10].

### 2.6. Statistics

The count data, as well as the categorical data, are reported as counts and percentages. Continuous data were initially assessed for normality using the Shapiro–Wilk test and by visual inspection of their histograms. Based on these results, the data are shown as medians [25%; 75% quartiles].

The Kruskal–Wallis test was initially used to test for the overall equality of medians in the dose parameters, as well as in subjective and objective image quality parameters between the CT scanner models. Single post-test comparisons were performed using Dunn’s test with the Bonferroni correction for multiple comparisons.

Analysis was performed using R (R Core Team (2022). R: A language and environment for statistical computing. R Foundation for Statistical Computing, Vienna, Austria. URL: https://www.R-project.org/, version 4.2.2, accessed on 15 April 2024). A level of significance of alpha = 0.05 was used throughout the study.

## 3. Results

A total of 1790 patients (883 female; 18 to 99 years, median age 78 [65; 84] years) with a total of 1864 examinations were included. In 1722 patients (96.2%), one examination was performed; 62 patients (3.5%) were examined twice, and in 6 patients (0.3%), three scans were conducted.

Most CT scans were performed with DSCT-1 (*n* = 1782, 95.6%), followed by DSCT-2 (*n* = 43, 2.3%), and SSCT (*n* = 39, 2.1%) (Figure 1). In detail, 687 (38.6%) and 1095 (61.4%) extended stroke protocols with and without CTP, respectively, were carried out by DSCT-1; 15 (34.9%) and 28 (65.1%) were performed by DSCT-2; and 10 (25.6%) and 29 (74.4%) were performed by SSCT.

### 3.1. Total Radiation Dose Analysis

The total DLP without CTP for all three CT scanner models was 903.4 [765.3; 3068.1] mGy∙cm, and the total ED was 1.65 [1.33; 4.99] mSv (Table 1). Additional CTP yielded a notable increase in the total DLP to 3579.2 [2953.0; 3708.3] mGy∙cm and in the total ED to 5.52 [4.71; 5.75] mSv, respectively.

**Figure 2:**
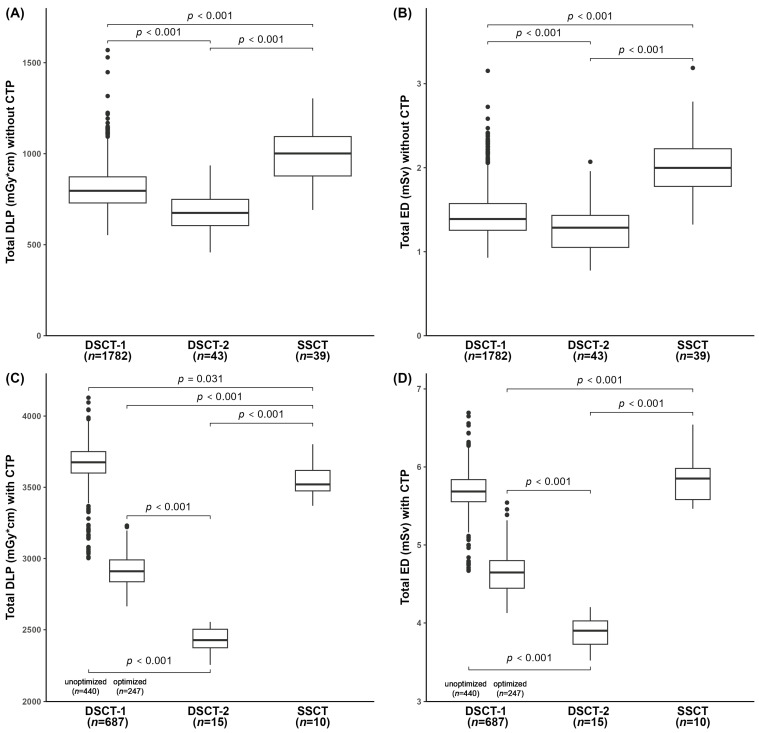
Total DLP and total ED of the extended stroke protocol among the three CT scanners without (**A**,**B**) and with CTP (**C**,**D**). For DSCT-1, total dosimetry values with CTP are shown before and after protocol optimization. cm, centimeter; CTP, computed tomography perfusion; DLP, dose length product; DSCT, dual-source computed tomography; ED, effective dose; mGy, milligray; mSv, millisievert; n, number of examinations; *p*, level of marginal significance; SSCT, single-source computed tomography. Horizontal bars represent significant intergroup differences.

In the group without CTP, the total dosimetrics were highest for SSCT, followed by DSCT-1, while the lowest values were recorded for DSCT-2. These differences were statistically significant (*p* < 0.001, each) (Figure 2A,B).

Considering additional CTP, the total radiation dose remained significantly lowest for DSCT-2 (*p* < 0.001 each; Figure 2C,D). The significantly (*p* ≤ 0.031) highest DLP was reached by DSCT-1 during the unoptimized period. The ED of DSCT-1 and SSCT scanners was statistically equal (*p* = 0.496), with the values for each being significantly (*p* < 0.001) higher compared to DSCT-2.

After CTP protocol optimization at DSCT-1, this device yielded significantly lower dosimetrics than SSCT (*p* < 0.001), while values remained significantly higher compared to DSCT-2 (*p* < 0.001).

### 3.2. Dose Analysis for Individual Sequences

The detailed dosimetry distribution for each sequence is presented in Supplementary Table S2.

For NCCT, the highest dosimetrics were measured for SSCT followed by DSCT-1, while the lowest dosimetrics were observed for DSCT-2. These intergroup differences were statistically significant (*p* < 0.001), except for DLP between SSCT and DSCT-1 (*p* = 0.120).

Regarding arterial CTA, SSCT yielded significantly higher dosimetrics compared to both DSCTs (*p* < 0.001, each). Dose values tended to be lower for DSCT-1 than for DSCT-2, without being statistically significant (*p* > 0.999).

The sequence parameters for early and late venous CTA were identical within the respective scanner models, with each yielding a similar dose distribution. All three scanners showed significant (*p* < 0.001) intergroup differences, with the highest and lowest dosimetry values for SSCT and DSCT-2, respectively.

Regarding CTP, after dose optimization in DSCT-1, this scanner yielded significantly higher and lower DLP/ED values than DSCT-2 and SSCT (*p* < 0.001, each), respectively. During the preceding unoptimized phase, both values were still the highest for DSCT-1 and differed significantly (*p* < 0.001) from those of DSCT-2 and SSCT.

CTP CTDIvol was consistently highest for SSCT. During the unoptimized phase, CTP CTDIvol for DSCT-1 was significantly higher and lower compared to DSCT-2 and SSCT (*p* < 0.001, each), respectively. After optimization, DSCT-1 yielded the lowest CTP CTDIvol, with significant difference compared to DSCT-2 (*p* < 0.001).

### 3.3. Objective Image Quality

The details of the objective image quality analysis for each CT phase are presented in Table 2.

Regarding NCCT, GM and WM noise values were significantly higher for SSCT compared to both DSCTs (*p* < 0.05, each). Accordingly, the respective SNRs were significantly lower (*p* < 0.020, each) when using SSCT.

**Figure 3:**
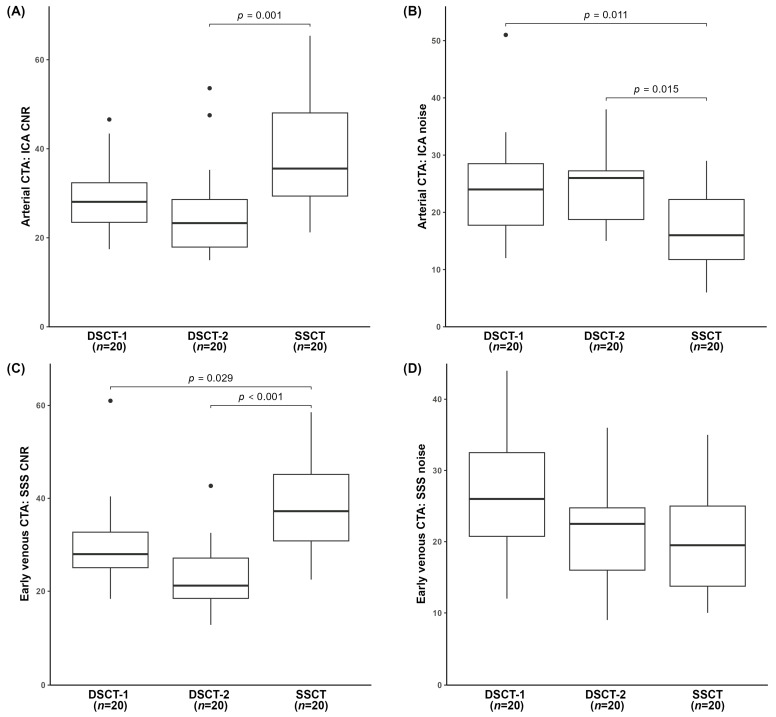
Objective image quality analysis of arterial CTA (**A**,**B**) and early venous CTA (**C**,**D**), showing the distribution of CNR and image noise among the three CT scanners. CNR, contrast-to-noise ratio; CTA, computed tomography angiography; DSCT, dual-source computed tomography; ICA, internal carotid artery; n, number of examinations; SSCT, single-source computed tomography; SSS, superior sagittal sinus. Horizontal bars represent significant intergroup differences.

The respective signal peak values were reached in the arterial and early venous CTA; thus, only these two phases are illustrated in Figure 3. In detail, for arterial CTA, SSCT yielded the highest ICA CNR, with a significant difference compared to DSCT-2 (*p* < 0.001) (Figure 3A). Likewise, ICA noise was significantly lower for SSCT than for both DSCTs (*p* ≤ 0.015, each) (Figure 3B).

During early venous CTA, ICA CNR was significantly higher at SSCT compared to DSCT-2 (*p* = 0.033). SSCT also yielded the lowest ICA noise, with the difference reaching statistical significance (*p* < 0.001) versus DSCT-1. SSS CNR exhibited significantly (*p* ≤ 0.029) higher values at SSCT compared to both DSCTs (Figure 3C). The lowest SSS CNR was recorded for DSCT-2 (*p* = 0.057) compared to DSCT-1. SSS noise tended to be lower for SSCT compared to both DSCTs (*p* > 0.054, Figure 3D).

The ICA and SSS CNRs during late venous CTA were the highest for SSCT and the lowest for DSCT-2, reaching statistical significance (*p* ≤ 0.013). Also, the respective noise values were significantly lower for SSCT compared to both DSCTs (*p* ≤ 0.032, each).

With regard to CTP, no significant differences in CNR and noise were calculated. However, ICA CNR (peak phase) tended to be lower for SSCT compared to both DSCTs (*p* < 0.316, each).

### 3.4. Subjective Image Quality

**Figure 4:**
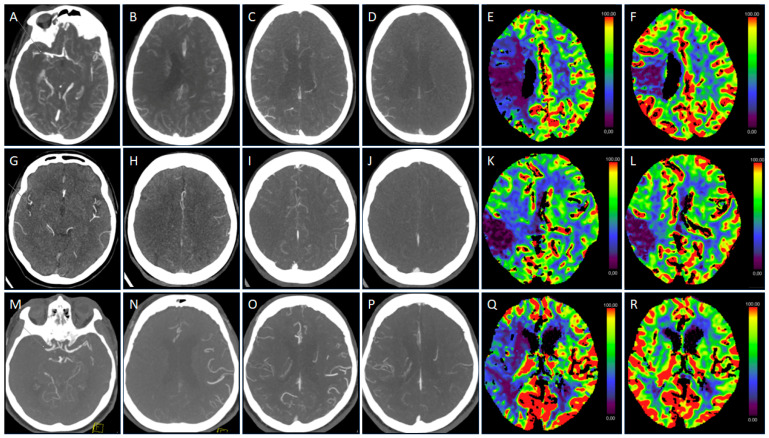
(**A**–**F**) Examples of MP-CTA and CTP results with the DSCT-1 scanner: (**A**) Arterial CTA phase shows an M1-MCA occlusion on the right (arrow). Axial MIP reconstructions of the arterial (**B**), early venous (**C**), and late venous phase (**D**) depict intermediate collaterals with the delayed pial filling of two phases in the central MCA territory and of one phase in the adjacent brain regions, respectively. Correspondingly, CTP shows a mismatch between CBF (**E**) and CBV (**F**), indicating an infarct core in the central MCA territory with surrounding tissues at risk. Overall image quality was rated excellent (median 4). (**G**–**L**) Examples of MP-CTA and CTP results using the DSCT-2 scanner. (**G**) Arterial CTA phase shows an insular M2-MCA occlusion on the right (arrow). Axial MIP reconstructions of the arterial (**H**), early venous (**I**), and late venous CTA (**J**) demonstrate poor collaterals in the central MCA territory with no pial filling in each phase. CTP illustrates a corresponding complete match between CBF (**K**) and CBV (**L**), indicating a distinct infarct core without surrounding tissues at risk. Image quality of MP-CTA and CTP was rated good (median 3 of 4 due to slightly increased noise) and excellent (median 4), respectively. (**M**–**R**): Examples of MP-CTA and CTP results with the SSCT scanner. (**M**) Arterial CTA phase shows a proximal M1-MCA occlusion on the right (arrow). Yellow graphic: Partially displayed orientation cube. Axial MIP reconstructions of the arterial (**N**), early venous (**O**), and late venous CTA (**P**) demonstrate intermediate collaterals with the delayed pial filling of one phase within the whole MCA territory. Yellow graphic: Partially displayed orientation cube. Similarly, CTP shows a major mismatch of the entire MCA territory between CBF (**Q**) and CBV (**R**), indicating a large volume of tissue at risk. Overall image quality was rated excellent (median 4). CBF, cerebral blood flow; CBV, cerebral blood volume; CTA, computed tomography angiography; CTP, CT perfusion; DSCT, dual-source CT; M1-MCA, M1 segment of the middle cerebral artery; MIP, maximum intensity projection; MP, multiphase.

The MP-CTA scores for DSCT-2 were slightly but significantly lower (*p* < 0.05, each), with a median [25%, 75% quartile] score of three [3; 3], compared to DSCT-1 and SSCT, each scoring four [4; 4]. The image quality of the remaining sequences (NCCT and CTP) did not differ significantly among scanner models and consistently provided a high diagnostic quality (median rating 4, each) (Figure 4).

## 4. Discussion

In this study, we provide detailed data on radiation dose and the image quality of the extended stroke protocol applied to three contemporary CT scanners (DSCT-1, 2 × 192 slices; DSCT-2, 2 × 128 slices; SSCT, 128 slices). We believe that our findings may be valuable for the expansion of the DRL catalog, while also considering the dynamic evolution of diagnostic and therapeutic approaches in AIS. Particularly, MP-CTA represents a powerful tool with a low burden of radiation dose that can be easily implemented into the radiological workflow.

Over the last decades, several new CT scanner generations and techniques have been introduced (e.g., multislice CT, DSCT with fully integrated detectors, AEC, IR, and high-pitch CT) [18,19,20,21,22,23,24], allowing for a continuous reduction in radiation exposure over time, while the image quality has increased substantially. This technical evolution is also reflected by nationally published DRLs representing gathered data from multiple institutions being regularly updated. Regarding stroke imaging, for example, in 2008, Yang and colleagues reported a CTDIvol of 51.72 mGy for SP-CTA and 623.31 mGy for CTP, respectively, using 64-slice SSCT while only allowing for a 4 cm CTP scan range [16]. In 2022, however, the German DRL for SP-CTA had already been reduced to 15 mGy [14]. Again, DRLs for MP-CTA and CTP have yet to be defined.

The current national DRLs for NCCT in Germany (55 mGy, [14]), as well as in several nations of the Commonwealth and the USA (47–82 mGy, [15]), were observed by all three scanners in this study, with dosimetrics being significantly lower for both DSCTs than for SSCT. Also, the median ED for NCCT was substantially lower compared to published data (this study, 0.78–1.09 mSv; Menon et al., 2.0 mSv; Wu et al., 1.83 mSv) [10,19].

More importantly, we each recorded an exceedingly low CTDIvol for individual MP-CTA scans, clearly below the abovementioned German DRL for SP-CTA (no respective DRLs have been defined in the other mentioned countries so far), with significantly highest values for SSCT. In detail, local DRLs for arterial CTA ranged between 3.6 mGy (DSCT-1) and 5.5 mGy (SSCT), while those values for early/late venous CTA each ranged between 1.2 mGy (DSCT-2) and 2.5 mGy (SSCT). Likewise, very low summed ED values were noted for MP-CTA (arterial CTA, range 0.32–0.75 mSv; early/late venous CTA, range 0.03–0.07 mSv, each) compared to other authors (Menon et al., arterial CTA 5.0 mSv, early/late venous CTA 1.0 mSv, each; Wu et al., arterial CTA 1.83 mSv) [10,19].

With regard to CTP, compared to the abovementioned data of Yang and colleagues [16], our recorded DRLs were substantially lower. In detail, the significantly lowest and highest DRLs were observed for DSCT-1 (141.1 mGy) and SSCT (220.5 mGy), respectively. However, one must still consider the notable dosimetry amount of CTP. In our study, for example, even though we implemented an optimized protocol for DSCT-1 (dose reduction 28.2%), CTP radiation exposure using this scanner still exceeded NCCT by a factor of three (median DLP 2087 versus 645 mGy∙cm, ED 3.13 versus 0.97 mSv). In comparison, the CTP of DSCT-2 yielded significantly lower dosimetrics (DLP 1748 mGy∙cm, ED 2.62 mSv), even though the distinct dose escalation versus NCCT persisted (DLP 517 mGy∙cm, ED 0.78 mSv).

The higher CTP DLP/ED values at DSCT-1 (versus DSCT-2) despite a lower CTDIvol were primarily due to its capability for whole-brain CTP, representing a unique feature in this study [20,21]. Thus, the dose escalation when using DSCT-1 seems reasonable, considering the obviously diagnostic advantage of whole-brain CTP, as even tiny perfusion deficits in regions that might otherwise be missed can be visualized. Nevertheless, the CTP ED values of both DSCTs were each clearly lower than those recorded by others (Menon et al., 3.5 mSv; Döring et al., 5.3 mSv) [10,17]. The CTP ED reported by Döring and colleagues even exceeded the summed value observed in this study for the entire stroke protocol using DSCT (DSCT-1, 4.65 mSv; DSCT-2, 3.9 mSv), while being only slightly lower than the summed ED of our SSCT (5.85 mSv).

Overall, the image quality for NCCT and MP-CTA was rated highly. Interestingly, we documented slightly increased noise (arterial/early venous CTA) when using DSCT-2, yielding a significantly lower median CNR compared to SSCT (arterial CTA, 23.3 versus 35.5; early venous CTA, 21.2 versus 37.2), which was also noted in the qualitative analysis. As mentioned above, the dosimetrics were in turn significantly higher when using SSCT. The CTA noise factor was also relatively high for DSCT-1, but the image quality was rated slightly higher compared to DSCT-2 due to the superior intravascular signal (CNR arterial/early venous CTA, 28.1/28.0). The moderately increased noise at both DSCTs can be explained by a relatively low AEC-triggered tube output, even though it did not substantially affect the diagnostic accuracy. To reduce image noise, an increase in tube output for MP-CTA at both DSCT scanners might be reasonable, considering the superior dosimetry profile of DSCT as (i) both DSCTs in this study are equipped with the Stellar detector, enabling signal transformation within the detector itself, which in turn allows for a 54% dose reduction [28], and (ii) the advanced modeled IR algorithm (ADMIRE) of both DSCTs yields superior image quality compared to the IR algorithm used with the SSCT [29]. Taking a look at the literature, however, our measured CNRs (arterial CTA) were still clearly higher for all three scanners (with the ED values each being lower) compared to data reported by Wu and colleagues, who applied a 256-slice SSCT (CNR 5.38, ED 1.83 mSv) [19].

Also, we each noted excellent CTP image quality despite reducing the fixed tube current time product for DSCT-1, with maximum CNRs ranging between 26 (SSCT) and 35.9 (DSCT-2). Thus, a further CTDIvol reduction in CTP might be achievable, e.g., when applying both lower tube output and IR, according to Niesten and colleagues [30], as well as Wu and colleagues [19]. In the latter study, an ultra-low-dose CTP protocol using 50 mAs (this study used 180–200 mAs) in combination with a dedicated knowledge-based iterative model reconstruction algorithm allowed for a 50% dose reduction (scan range, 8 cm; ED, 1.28 mSv) [19]. Another technique for CTP dose optimization is the usage of the temporal information of preceding CTA bolus tracking, as described by Deak and colleagues [31], which used a similar DSCT-1 to that in our study. By applying their technical approach, the number of head scans needed for CTP acquisition could be reduced to a minimum of *n* = 20 (this study used DSCT with *n* = 32 and SSCT with *n* = 30), allowing for a 37.5% dose reduction without any loss of image quality. Conversely, CTP raw data may also be used to virtually calculate MP-CTA data in order to (i) skip the otherwise needed double contrast injection and (ii) reduce the overall radiation dose [19]. Furthermore, recent data suggest that recurrent neural networks [32] and photon-counting CT [33] harbor novel possibilities for optimizing radiation exposure and image quality.

The scope of this study included the dosimetry and image quality analysis of an extended stroke CT protocol with and without CT perfusion; we thus did not consider the clinical outcomes of our patient cohort. However, taking into account the bigger picture of contemporary stroke imaging and therapy, recent randomized–controlled trials have proven the clinical benefit of mechanical thrombectomy in large-core AIS-LVO patients [34,35]. Thus, CTP may no longer be mandatory in these patients, as they still qualify for endovascular revascularization. Nevertheless, CTP remains valuable in selected patients (e.g., to differentiate between distal vessel occlusion yielding tiny perfusion deficits and AIS mimics such as seizure or encephalitis). To summarize, regarding the potential clinical pathway of the extended stroke protocol, in suspected AIS patients, we recommend (1) NCCT to exclude intracranial bleeding for potential intravenous lysis therapy, (2) MP-CTA, which is superior to SP-CTA regarding LVO/MVO detection, and (3) additional CTP only in the case of a mismatch between the clinical symptoms and imaging findings (e.g., a severe neurological deficit but negative NCCT/MP-CTA) to narrow down potential differential diagnoses, as mentioned above.

The limitations of our study include (i) its single-center design; (ii) the use of three CT scanners from only one specific manufacturer (Siemens Healthineers), which differ substantially in terms of hardware, software, and protocol settings (e.g., X-ray tubes, detector technology, and IR algorithms)—in particular, the dosimetric comparison of CTP between devices is difficult due to the different acquisition modes (number of scans, field of view); and (iii) the relatively small sample sizes for DSCT-2 (CTP *n* = 15) and SSCT (CTP *n* = 10).

## 5. Conclusions

In conclusion, we assessed local DRLs for the extended stroke protocol, including novel DRLs for additive MP-CTA scans and CTP applied using three different CT scanners, providing a useful expansion of the DRL catalog. In detail, the calculated local DRLs for arterial CTA ranged between 3.6 and 5.5 mGy; for early/late venous CTA, they ranged between 1.2 and 2.5 mGy; and for CTP, they ranged between 141.1 and 220.5 mGy. Both DSCTs allowed for significantly lower radiation exposure compared to SSCT, with local DRLs for NCCT and individual MP-CTA scans each clearly remaining below national DRLs for NCCT and SP-CTA. CTP protocol adjustment using a third-generation DSCT yielded a 28.2% reduction in effective dose. The objective and subjective image quality was high for all three scanners. For the definition of national DRLs, however, we recommend prospective multi-center studies gathering data derived from the extended stroke CT protocol, also considering different manufacturers and modern dose reduction techniques.

## Figures and Tables

**Table 1:** Total radiation dose of the extended stroke CT protocol.

	Summed dosimetrics of the whole study population (all CT scanners)						
NCCT + MP-CTA (without CTP)							
DLP (mGy∙cm)	903.4 [765.3; 3068.1] ^1^						
ED (mSv)	1.65 [1.33; 4.99] ^1^						
NCCT + MP-CTA (with CTP)							
DLP (mGy∙cm)	3579.2 [2953.0; 3708.3] ^2^						
ED (mSv)	5.52 [4.71; 5.75] ^2^						
	Summed dosimetrics for each CT scanner						
	Scanner model				*p* Value ^†^		
	DSCT-1	^#^	DSCT-2	SSCT	DSCT-1 vs. DSCT-2	DSCT-1 vs.SSCT	DSCT-2 vs. SSCT
NCCT + MP-CTA (without CTP)							
DLP (mGy∙cm) *	796.1 [729.3; 872.8] ^3^		674.7 [605.1; 749.1] ^6^	1001.3 [877.7; 1094.1] ^8^	**<0.001**	**<0.001**	**<0.001**
ED (mSv) *	1.39 [1.25; 1.57] ^3^		1.29 [1.05; 1.43] ^6^	2.00 [1.78; 2.23] ^8^	**<0.001**	**<0.001**	**<0.001**
NCCT + MP-CTA (with CTP)							
DLP (mGy∙cm) *	3675.5 [3600.1; 3750.5] ^4^	u	2427.1 [2373.6; 2503.3] ^7^	3520.3 [3474.8; 3618.5] ^9^	**<0.001**	**0.031**	**<0.001**
	2910.5 [2836.9; 2990.4] ^5^	o			**<0.001**	**<0.001**	**<0.001**
ED (mSv) *	5.69 [5.55; 5.84] ^4^	u	3.90 [3.73; 4.03] ^7^	5.85 [5.58; 5.98] ^9^	**<0.001**	0.496	**<0.001**
	4.65 [4.45; 4.80] ^5^	o			**<0.001**	**<0.001**	**<0.001**

CT, computed tomography; CTP, CT perfusion; DLP, dose length product; DSCT, dual-source CT; ED, effective dose; mGy, milligray; MP-CTA, multiphase CT angiography; NCCT, non-contrast CT; mSv, millisievert; SSCT, single-source CT. Data are presented as median [25% quartile; 75% quartile (diagnostic reference level)]. * Significant intergroup difference detected with Kruskal–Wallis analysis (*p* < 0.001). ^†^ Values in bold indicate significant intergroup differences with post hoc comparisons (Dunn’s test). Number of examinations: ^(1)^ *n* = 1864; ^(2)^ *n* = 712; ^(3)^ *n* = 1782; ^(4)^ *n* = 440; ^(5)^ *n* = 247; ^(6)^ *n* = 43; ^(7)^ *n* = 15; ^(8)^ *n* = 39; ^(9)^ *n* = 10. ^(#)^ Period of CT perfusion protocol on the DSCT-1 scanner: u, unoptimized; o, optimized.

**Table 2:** Objective image quality analysis of extended stroke CT protocol.

	Scanner Model			*p* Value ^†^		
	DSCT-1	DSCT-2	SSCT	DSCT-1 vs. DSCT-2	DSCT-1 vs. SSCT	DSCT-2 vs. SSCT
NCCT						
GM _SNR_ *	14.3 [11.0; 14.7] ^1^	13.8 [11.2; 14.7] ^1^	10.0 [9.1; 10.8] ^1^	>0.999	**<0.001**	**<0.001**
GM _noise_ **	3.0 [3.0; 4.0] ^1^	3.0 [3.0; 4.0] ^1^	4.0 [4.0; 4.3] ^1^	>0.999	**0.002**	**0.001**
WM _SNR_ **	10.7 [9.6; 11.1] ^1^	11.0 [9.5; 11.8] ^1^	8.3 [7.8; 9.8] ^1^	>0.999	**0.020**	**0.001**
WM _noise_ ***	3.0 [3.0; 3.3] ^1^	3.0 [3.0; 3.3] ^1^	4.0 [3.0; 4.0] ^1^	>0.999	**0.020**	**0.006**
Arterial CTA						
ICA _CNR_ ***	28.1 [23.5; 32.4] ^1^	23.3 [17.9; 28.6] ^1^	35.5 [29.4; 48.0] ^1^	0.474	0.089	**0.001**
ICA _noise_ ***	24.0 [17.8; 28.5] ^1^	26.0 [18.8; 27.3] ^1^	16.0 [11.8; 22.3] ^1^	>0.999	**0.011**	**0.015**
SSS _CNR_ *	15.4 [12.2; 19.2] ^1^	5.8 [4.1; 8.6] ^1^	14.6 [10.9; 18.2] ^1^	**<0.001**	>0.999	**<0.001**
SSS _noise_ *	19.5 [15.0; 26.5] ^1^	14.0 [10.8; 21.0] ^1^	8.0 [6.8; 10.3] ^1^	0.137	**<0.001**	**0.001**
Early venous CTA						
ICA _CNR_	13.6 [10.1; 20.4] ^1^	13.3 [7.9; 17.7] ^1^	20.7 [15.1; 27.8] ^1^	>0.999	0.076	**0.033**
ICA _noise_ *	23.5 [18.3; 27.3] ^1^	19.0 [12.5; 22.0] ^1^	13.0 [10.0; 15.0] ^1^	0.094	**<0.001**	0.076
SSS _CNR_ *	28.0 [25.1; 32.7] ^1^	21.2 [18.5; 27.1] ^1^	37.2 [30.8; 45.1] ^1^	0.057	**0.029**	**<0.001**
SSS _noise_	26.0 [20.8; 32.5] ^1^	22.5 [16.0; 24.8] ^1^	19.5 [13.8; 25.0] ^1^	0.191	0.054	>0.999
Late venous CTA						
ICA _CNR_ ****	7.5 [5.6; 9.2] ^1^	5.0 [3.5; 7.8] ^1^	9.6 [6.9; 11.0] ^1^	0.518	0.420	**0.013**
ICA _noise_ *	20.5 [18.5; 26.0] ^1^	17.5 [16.0; 22.5] ^1^	11.5 [9.0; 15.3] ^1^	>0.999	**<0.001**	**0.001**
SSS _CNR_ ***	19.8 [15.5; 22.6] ^1^	14.5 [9.8; 21.1] ^1^	25.0 [18.8; 28.8] ^1^	0.686	0.093	**0.002**
SSS _noise_ ***	17.0 [14.0; 25.0] ^1^	19.0 [16.5; 23.5] ^1^	11.5 [10.0; 17.3] ^1^	>0.999	**0.032**	**0.012**
CTP (arterial peak)						
ICA _CNR_	31.8 [25.9; 47.4] ^2^	35.9 [26.4; 41.8] ^3^	26.0 [18.8; 33.0] ^4^	>0.999	0.192	0.316
ICA _noise_	22.0 [16.5; 28.0] ^2^	26.0 [16.0; 28.5] ^3^	22.5 [16.5; 28.3] ^4^	>0.999	>0.999	>0.999

CNR, contrast-to-noise ratio; CT, computed tomography; CTA, computed tomography angiography; CTP, CT perfusion; DSCT, dual-source CT; GM, gray matter; WM, white matter; ICA, internal carotid artery; SSS, superior sagittal sinus; SNR, signal-to-noise ratio; NCCT, non-contrast CT; SSCT, single-source CT. Data are presented as median [25% quartile; 75% quartile]. Significant intergroup difference detected with Kruskal–Wallis analysis: ^(^*^)^ *p* < 0.0001; ^(^**^)^ *p* < 0.001; ^(^***^)^ *p* < 0.01; ^(^****^)^ *p* < 0.05. ^(†)^ Values in bold indicate significant intergroup difference with post hoc comparisons. Number of examinations: ^(1)^ *n* = 20; ^(2)^ *n* = 40; ^(3)^ *n* = 15; ^(4)^ *n* = 10.

## Data Availability

The data presented in this study are available upon reasonable request from the corresponding author.

## Supplementary Materials

The following supporting information can be downloaded at the following website: https://www.mdpi.com/article/10.3390/diagnostics14242866/s1, Table S1: Protocol settings for extended stroke CT; Table S2: Radiation dose for extended stroke CT protocol for each sequence.
