# Supplementary material for: Optimizing Radiation Dose and Image Quality in Stroke CT Protocols: Proposed Diagnostic Reference Levels for Multiphase CT Angiography and Perfusion Imaging
Source: Diagnostics (Basel). 2024 Dec 20;14(24):2866. doi: 10.3390/diagnostics14242866 (PMC11675730; doi:10.3390/diagnostics14242866)
Supplement: Supplementary file 1 [file diagnostics-14-02866-s001.zip › diagnostics-3352068-supplementary.pdf]

diagnostics

MDPI

Supplementary Table S1: Protocol settings of extended stroke CT

	Scanner Model		
	**DSCT-1**	**DSCT-2**	**SSCT**
**NCCT**			
Scan range		Skull base to vertex	
Iterative reconstruction	ADMIRE	ADMIRE	SAFIRE
Slice thickness [mm]	0.75	0.75	1
Reconstruction thickness [mm)	5	5	5
Scan time [s]	15.95	20	20
Rotation time [s]	1	1	1
Pitch	0.55	0.55	0.55
AEC		CARE Dose 4D, Care kV	
**Arterial CTA**			
Scan range		Aortic arch to vertex	
Iterative reconstruction	ADMIRE	ADMIRE	SAFIRE
Slice thickness [mm]	0.75	0.75	0.75
MIP thickness [mm]	10	10	10
Scan time [s]	4.01	2.27	3.19
Rotation time [s]	0.25	0.28	0.5
Pitch	1.2	1.2	1.5
AEC		CARE Dose 4D, Care kV	
Contrast [mg I/ml]		50 ml Imeron 400	
Delay [s]	7	2	5
**Early/late venous CTA**			
Scan range		Skull base to vertex	
Iterative reconstruction	ADMIRE	ADMIRE	SAFIRE
Slice thickness [mm]	0.75	0.75	0.75
MIP thickness [mm]	10	10	10
Scan time [s]	2.02	2.27	3.19
Rotation time [s]	0.25	0.28	0.5
Pitch	1.2	1.2	1.5
AEC		CARE Dose 4D, Care kV	
Delay, each [s]	8	8	8
**CTP**			
Scan length [mm]	201	114	118
Slice thickness [mm]	1.5	1.5	1.5
Scan time [s]	1.5	1.5	1.5
Rotation time [s]	0.25	0.28	0.3
Contrast [mg I/ml]		40 ml Imeron 400	

Delay [s]	8	2	6
Tube current time product [mAs]	250[u]/180[o]	200	200
Tube voltage [kV]	70	70	80
Exam time [s]	48.48	49.44	46.35
No. of scans	32	32	30

AEC: Automatic exposure control; CT: Computed tomography; CTA: Computed tomography angiography; CTP: CT perfusion; DSCT: Dual-source CT; 4D: Four-Dimensional; kV: kilovolt; cm: centimeter; MIP: maximum intensity projection; mm: millimeter; mA: milliamp; mAs: milliamp second; ml: milliliters; NCCT: non-contrast CT; No.: number; s: second; ADMIRE: Advanced modeled iterative reconstruction; SAFIRE: Sinogram affirmed iterative reconstruction; SSCT: Single-source CT; u: unoptimized CT perfusion protocol; o: optimized CT perfusion protocol; CARE Dose4D: Manufacturer specific name for the AEC method.

Supplementary Table S2: Radiation dose of extended stroke CT protocol for each sequence

	Scanner Model				*P* Value †		
	**DSCT-1**	#	**DSCT-2**	**SSCT**	DSCT-1 vs. DSCT-2	DSCT-1 vs. SSCT	DSCT-2 vs. SSCT
**NCCT**							
$CTDI_{vol}$ (mGy)*	42.0 [39.4; **44.8**] [1]		35.5 [32.7; **37.3**] [2]	45.0 [42.4; **49.1**] [3]	**<0.001**	**<0.001**	**<0.001**
DLP (mGy·cm)*	644.8 [594.7; 697.7] [1]		517.0 [488.1; 545.2] [2]	682.7 [612.7; 734.4] [3]	**<0.001**	0.120	**<0.001**
ED (mSv)*	0.97 [0.89; 1.05] [1]		0.78 [0.73; 0.82] [2]	1.09 [0.98; 1.17] [3]	**<0.001**	**<0.001**	**<0.001**
**Arterial CTA**							
$CTDI_{vol}$ (mGy)*	2.4 [1.6; **3.6**] [1]		3.5 [1.7; **3.8**] [2]	5.3 [4.9; **5.5**] [3]	>0.999	**<0.001**	**<0.001**
DLP (mGy·cm)*	87.3 [63.7; 133.4] [1]		131.6 [65.7; 152.8] [2]	209.7 [186.1; 252.8] [3]	>0.999	**<0.001**	**<0.001**
ED (mSv)*	0.32 [0.23; 0.49] [1]		0.47 [0.24; 0.55] [2]	0.75 [0.67; 0.91] [3]	>0.999	**<0.001**	**<0.001**
**Early venous CTA**							
$CTDI_{vol}$ (mGy)*	1.6 [1.5; **1.6**] [1]		1.1 [1.1; **1.2**] [2]	2.5 [2.4; **2.5**] [3]	**<0.001**	**<0.001**	**<0.001**
DLP (mGy·cm)*	25.4 [24.2; 26.8] [1]		19.6 [18.4; 22.6] [2]	46.5 [44.3; 48.9] [3]	**<0.001**	**<0.001**	**<0.001**
ED (mSv)*	0.04 [0.04; 0.04] [1]		0.03 [0.03; 0.03] [2]	0.07 [0.07; 0.07] [3]	**<0.001**	**<0.001**	**<0.001**
**Late venous CTA**							
$CTDI_{vol}$ (mGy)*	1.6 [1.5; **1.6**] [1]		1.1 [1.1; **1.2**] [2]	2.5 [2.4; **2.5**] [3]	**<0.001**	**<0.001**	**<0.001**
DLP (mGy·cm)*	25.4 [24.2; 26.9] [1]		19.6 [18.4; 21.5] [2]	46.8 [43.9; 49.1] [3]	**<0.001**	**<0.001**	**<0.001**
ED (mSv)*	0.04 [0.04; 0.04] [1]		0.03 [0.03; 0.03] [2]	0.07 [0.07; 0.07] [3]	**<0.001**	**<0.001**	**<0.001**
**CTP**							
$CTDI_{vol}$ (mGy)*	196.1 [195.8; 196.1] [4]	u	148.2 [147.9; **148.4**] [6]	220.5 [219.8; **220.5**] [7]	**<0.001**	**<0.001**	**<0.001**
	140.8 [140.8; **141.1**] [5]	o			**<0.001**	**<0.001**	>0.999
DLP (mGy·cm)*	2908.2 [2902.5; 2908.4] [4]	u	1747.5 [1744.0; 1750.2] [6]	2511.5 [2503.9; 2511.5] [7]	**<0.001**	**<0.001**	>0.999
	2086.6 [2086.5; 2086.6] [5]	o			**<0.001**	**<0.001**	**<0.001**
ED (mSv)*	4.36 [4.35; 4.36] [4]	u	2.62 [2.62; 2.63] [6]	3.77 [3.76; 3.77] [7]	**<0.001**	**<0.001**	>0.999
	3.13 [3.13; 3.14] [5]	o			**<0.001**	**<0.001**	**<0.001**

CT: Computed tomography; CTA: Computed tomography angiography; $CTDI_{vol}$: Volumetric computed tomography dose index; DLP: Dose length product; DSCT: Dual-source CT; ED: Effective dose; Gy: Gray; NCCT: non-contrast CT; SSCT: Single-source CT; Sv: Sievert.

Data are median [25% quartile; 75% quartile (Diagnostic reference level)].

*Significant intergroup difference with Kruskal-Wallis analysis ($p<0.001$).† Values in bold indicate significant intergroup difference with post hoc comparisons (Dunn test).

Number of examinations: 1) n=1782; 2) n=43; 3) n=39; 4) n=440; 5) n=247; 6) n=15; 7) n=10.

#) Period of CT perfusion protocol on the DSCT-1 scanner: u: unoptimized, optimized

Bold $CTDI_{vol}$ values represent the local diagnostic reference value (75% percentile)
